# Current status of miRNA-targeting therapeutics and preclinical studies against gastroenterological carcinoma

**DOI:** 10.1186/2052-8426-1-5

**Published:** 2013-12-13

**Authors:** Chikako Shibata, Motoyuki Otsuka, Takahiro Kishikawa, Takeshi Yoshikawa, Motoko Ohno, Akemi Takata, Kazuhiko Koike

**Affiliations:** Department of Gastroenterology, Graduate School of Medicine, The University of Tokyo, Tokyo, 113-8655 Japan; Japan Science and Technology Agency, PRESTO, Kawaguchi, Saitama, 332-0012 Japan

**Keywords:** Locked nucleic acids, Miravirsen, MicroRNA, Therapeutics

## Abstract

Expanding knowledge about the crucial roles of microRNAs (miRNAs) in human diseases has led to the idea that miRNAs may be novel, promising therapeutic targets against various pathological conditions. The recent success of a human clinical trial using anti-miR-122 oligonucleotides against chronic hepatitis C virus has paved the way for this approach. In this review, we summarize briefly the current status of clinical trials of miRNA-targeting therapy and several representative preclinical trials against hepato-gastrointestinal carcinoma. In addition, we describe the currently available technologies for modification and delivery of oligonucleotides, which are essential in providing efficient, specific and safe approaches to targeting miRNAs.

## Introduction

The expression and functional importance of non-coding RNAs (ncRNAs), such as long ncRNAs and microRNAs, in various human diseases has been reported extensively [1–3]. Accordingly, clinical applications of ncRNAs are highly anticipated [4].

MicroRNAs (miRNAs) are small non-coding RNAs first discovered in *C. elegans*[5]. They are now known to be expressed in most organisms from plants to vertebrates [6]. Many miRNAs are functionally important, acting as oncogenes, tumor suppressors and crucial modulators in intracellular pathways [7].

miRNAs are generated from endogenous transcripts through maturation processing. Long primary miRNAs (pri-miRNAs) ranging in size from several hundred nucleotides (nt) to several kilobases are transcribed from the genome [8]. These are processed into stem-loop precursor miRNAs (pre-miRNAs) of ~70 nt by a ribonuclease III (RNase III) known as Drosha and DGCR8/Pasha in the nucleus [8–11]. After these processing steps, pre-miRNAs are transported to the cytoplasm via exportin-5 [12], where they are recognized by an RNA-induced silencing complex (RISC). This complex is composed of the RNase III Dicer, a double-strand RNA binding protein TRBP, and Argonaute2 (Ago2). Pre-miRNAs are cleaved into mature miRNAs of ~22 nt by Dicer [13]. The two RNA strands are separated, and the guide strand for the target mRNA remains associated with Ago2. RISC recognizes the target mRNA based on the complementarity between the guide miRNA and the mRNA transcript [14] within the 3′ untranslated region (UTR) [15]; the target mRNA is subsequently degraded or translationally inhibited [14, 16], resulting in post-transcriptional gene silencing [14]. While the molecular mechanisms for gene silencing by miRNA-mRNA targeting require further elucidation, some reports have suggested that miRNAs may sequester target mRNAs into P-bodies [17], where mRNA decay occurs through the initiation of rapid deadenylation [18] or translational repression occurs by inhibiting the binding of ribosomes to the 5′ caps of miRNAs [19].

Alterations in miRNA expression levels contribute to the pathogenesis of human malignancies. The changes result from various mechanisms, including deletions, amplifications or mutations at miRNA loci, epigenetic silencing, dysregulation of transcription factors that are related to the transcription of specific miRNAs, environmental factors such as cigarette smoke and infection, and gene polymorphisms [20–22]. Describing specific patterns of miRNA expression levels may be useful for diagnosis, prognosis or evaluating therapeutic response, or in miRNA-targeted therapies that repress or facilitate expression of specific miRNAs.

Many excellent reviews have discussed the aberrant expression and potential biological roles of miRNAs in gastroenterological diseases [23–28]. Here, we focus on the recent progress of clinical trials of miRNA-target therapies and representative preclinical trials against gastroenterological carcinoma; additionally, we outline the future work required for clinical utilization of miRNAs.

## Review

### Current clinical applications of miRNA-targeting therapeutics

#### Anti-miR-122 therapy against chronic hepatitis C

Experiments *in vitro* and *in vivo* have led to the development of potential new therapies targeting miRNAs. While targeting miRNAs in human clinical trials has focused largely on miRNA signatures as biomarkers for the diagnosis, prognosis, or therapeutic response to traditional treatment [22], two human clinical trials have assessed directed miRNA-targeting as therapeutics, according to ClinicalTrials.gov (http://clinicaltrials.gov) (Table 1). Both trials are related to gastroenterological diseases.

**Table 1:** **Current clinical applications targeting miRNAs in human**

miRNA	NIH identifier	Drug	Subjects	Outline/purposes	Reference
miR-122	NCT01646489	Miravirsen Telaprevir	Hapatitis C chronic hepatitis C	To assess the safety, tolerability, and affect on blood levels of miravirsen and telaprevir when co-administered miravirsen and telaprevir in healthy subjects. <Phase 1>	
	NCT01872936	Miravirsen Telaprevir Ribavirin	Chronic hepatitis C (genotype1) Null responders to treatment with peg IFNα/RBV therapy.	To assess the safety, tolerability, antiviral activity, genotype resistance associated with virological failure, pharmacokinetics and pharmacodynamics of two dose regimens of miravirsen in combination with telaprevir and ribavirin in subjects with hepatitis C virus genotype 1 infection. <Phase 2>	
	NCT01200420	Miravirsen	Hepatitis C	1. Determining the safety and tolerability of multiple dosing of miravirsen in subjects infected with chronic hepatitis C.	[29]
		Saline			
				2. Assessing of pharmacokinetics of miravirsen and assessment of miravirsen's effect on HCV viral titer. <Phase 2>	
	NCT01727934	Miravirsen	Hepatitis C (genotype1) Null responders to treatment with peg IFNα/RBV therapy.	To aseess the safety, antiviral activity, and pharmacokinetics of 9 subcutaneous injections of miravirsen monotherapy over a total of 12 weeks of treatment.<Phase 2>	
	NCT00688012	SPC3649	Hepatitis C	A placebo-controlled, double-blind, randomized, single dose, dose escalating trial in healthy men to evaluate the safety, tolerability, pharmacokinetics and pharmacodynamics of SPC3649. <Phase 1>	[30]
miR-34	NCT01829971	MRX-34	Primary HCC metastatic liver cancer	Evaluating the safety of MRX34 in patients with primary liver cancer or those with liver metastasis from other cancers. <Phase 1>	

SPC3649: the active component of miravirsen.

One of the most extensively studied miRNAs is miR-122, an abundant liver-specific miRNA that plays a critical role in liver function, such as fatty acid and cholesterol metabolism, and in the pathophysiology of liver diseases, such as hepatitis C viral (HCV) replication [11, 31–33]. Inhibition of miR-122 with locked-nucleic-acid (LNA)-based anti-miR-122 oligonucleotides complementary to miR-122, caused a long-lasting decrease in total plasma cholesterol in mice [31] and in monkeys [34]. Lanford *et al.* demonstrated that LNA-based anti-miR-122 oligonucleotides led to the long-lasting suppression of HCV viremia and improvement of HCV-induced liver pathology in chimpanzees [33]. In these three cases, no LNA-associated toxicity or histopathological changes were found in mice and non-human primates after short-term administration of the oligonucleotides [31, 33, 34]. These reports indicate that LNA-based anti-miRs can achieve efficient silencing of endogenous miRNA function in mammals and primates, which supports the application of anti-miRNA therapy to other human diseases.

**Figure 1:**
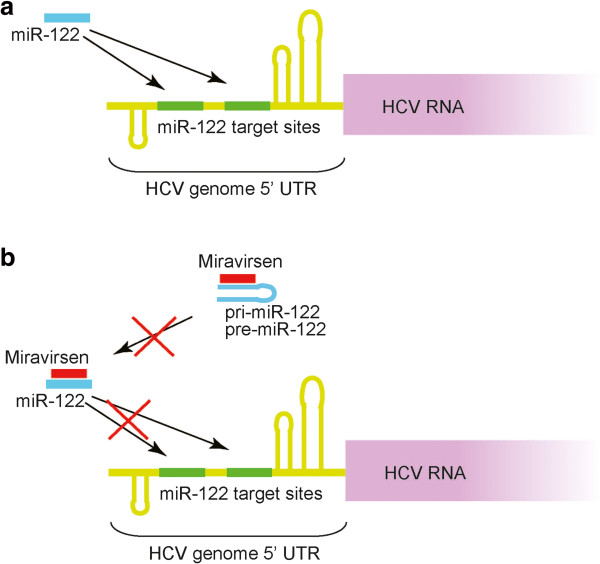
**Miravirsen inhibits miR-122. a**, Mir-122 binds two target sites in the HCV 5′ non-coding region and promotes HCV propagation. **b**, Miravirsen, a modified oligonucleotide complementary to miR-122 sequences, binds and sequesters mature miR-122, resulting in the functional inhibition of miR-122. Miravirsen also binds to the stem-loop structure of pri- and pre-miR-122 and inhibits the maturation of miR-122.

These preclinical results led to the development of miravirsen, a LNA-modified DNA phosphorothioate antisense oligonucleotide against miR-122, as the first miRNA-targeting drug for clinical use [29]. It was developed to target HCV since the stability and propagation of HCV is dependent on a functional interaction between the HCV genome and miR-122 [35]. miR-122 binds to two closely spaced target sites in the highly conserved 5′-untranslated region of the HCV genome, thereby forming an oligomeric miR-122-HCV complex that protects the HCV genome from nucleolytic degradation or from host innate immune responses [35]. The miR-122 binding sites are conserved across all HCV genotypes and subtypes [36]. Miravirsen, an LNA-modified DNA phosphorothioate oligonucleotide complementary to miR-122, is thought to hybridize to the 5′ region of mature miR-122, resulting in sequestration and inhibition of miR-122 [29]. Recently, it was reported that Miravirsen also binds to the stem-loop structure of pri- and pre- miR-122 and inhibits both Dicer- and Drosha- mediated processing of miR-122 precursors [30] (Figure 1).

No harmful events were observed in phase I studies of miravirsen in healthy volunteers; therefore, phase II studies were initiated to evaluate the safety and efficacy of miravirsen in 36 patients with chronic HCV genotype 1 infection. The patients were randomly assigned to receive 5-week subcutaneous injections of placebo or doses of miravirsen at 3, 5 or 7 mg per kilogram of body weight over a 29-day period. Patients who received miravirsen showed a dose-dependent reduction in HCV levels, without major adverse events and with no escape mutations in the miR-122 binding sites of the HCV genome [29].

The success of miravirsen is promising, not only as a novel anti-HCV drug, but also as the first trial of miRNA-targeting therapy. However, caution should be used since miR-122 is generally known as a tumor-suppressive miRNA. Downregulation of miR-122 expression in hepatocellular carcinoma (HCC) is associated with a poor prognosis [37–39], and liver tumorigenesis is facilitated in mice lacking miR-122 [40, 41]. Nonetheless, as an anti-HCV drug, short-term administration of miravirsen with a 4-week regimen was reversible, and the effects of a 12-week regimen were tested from November 2012–May 2013 (ClinicalTrials.gov Identifier: NCT01727934). Although miravirsen also showed promise for decreasing serum cholesterol levels, we cannot conclude that miravirsen remains free of adverse effects for long-term administration until a long-term trial is completed.

#### miR-34 mimics as a therapeutic against primary and metastatic liver cancer

In addition to miravirsen, a clinical trial of MRX34 as a mimic of miR-34 is ongoing. MRX34 is a liposome-formulated mimic of the tumor suppressor miR-34 (Mirna Therapeutics, Austin, TX). The expression levels of miR-34 are decreased in most human cancers [42–44], including several epithelial cancers, melanomas, neuroblastomas, leukemias and sarcoma [45]. miR-34 is involved in regulating the p53 pathway and inhibits cancer cell growth by directly targeting oncogenes such as Myc, c-Met, Bcl-2, CDK4, CDK6, Cyclin D1, and Cyclin E2 [42, 43, 46]. Liu *et al.* evaluated the survival of mice with established prostate tumors that received a miR-34 injection [43]. The authors reported that miR-34a extended the survival of tumor-bearing mice compared to mice that did not receive a miR-34 injection. Hu et al. demonstrated that systemic administration of a miR-34a delivery system in a pancreatic xenograft cancer model significantly inhibited tumor growth and induced cancer cell apoptosis [47]. Further study of MRX34 is being conducted by Mirna Therapeutics, which initiated a Phase I study in May 2013 to examine the effects of MRX34 on unresectable primary liver cancer or advanced or metastatic cancer with liver involvement (ClinicalTrials.gov Identifier: NCT01829971).

### Representative preclinical in vivo experiments

**Figure 2:**
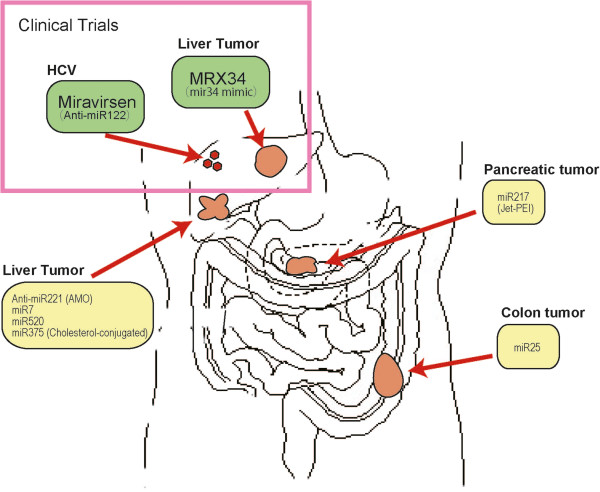
**Representative clinical and preclinical trials targeting miRNAs.** Currently on-going clinical trials and representative preclinical studies targeting miRNAs against cancers in the gastroenterological field.

Since their discovery, many novel miRNAs have been identified. As of September 2013, 2,578 mature human miRNA sequences were deposited in miRBase, a public repository hosted by the Sanger Institute (Cambridge, UK). miRNAs have diverse biological functions as they can target mRNAs with weak complementarity. Thus, it is not surprising that most miRNAs are associated with facilitating or suppressing tumors via modulating the expression levels of various tumor-related genes. The PubMed database contains nearly 25,000 miRNA-related articles, many of which aimed to identify novel targets of miRNAs, novel miRNAs, or novel functions of miRNAs in oncogenic/tumor suppressive function or stabilization. The majority of these *in vitro* studies need to be confirmed in mice or in non-human primates before their use in clinical trials. In the following section, we describe representative *in vivo* approaches to targeting miRNAs in hepato-gastroenterological cancers, which have evaluated the effects on the initiation, proliferation or growth of tumors in transgenic mice or an *in vivo* xenograft cancer model (Figure 2 and Table 2).

**Table 2:** **Representative preclinical in vivo experiments**

miRNA	Cancer	Target	Result	Reference
miR-221	HCC	CDKN1B/p27, CDKN1C/p57, Bmf, PTEN, TIMP3, DDIT4, mTOR	A transgenic mouse model of miR-221 overexpression in the liver was established, which is characterized by the inevitable appearance of spontaneous liver tumors with diethylnitrosamine. When received an *in vivo* intravenous injection of anti-miR-221 oligonucleotides exhibited a significant reduction in the number and size of liver tumor nodules.	[48]
miR-7	HCC	PIK3CD	In a xenograft model, overexpressed miR-7 effectively repressed tumor growth and decreased metastasis to the lung.	[49]
miR-520e	HCC	NIK	HepG2 cells transfected with miR-520e or a negative control were injected subcutaneously into nude mice. The introduction of miR-520e led to a significant reduction in both the size of tumor volume and the frequency of tumor formation. In addition, direct intratumoral injection with miR-520e oligonucleotides repressed the growth of HCC cells in an *in vivo* xenograft model.	[50]
miR-375	HCC	AEG-1	Overexpression of miR-375 in liver cancer cells decreased cell proliferation, clonogenicity, migration, and invasion, and induced G1 cell cycle arrest and apoptosis. Direct administration of cholesterol-conjugated 2’-O-methyl-modified miR375 mimics significantly affected the growth of HCC xenografts.	[51]
miR-25	Colon cancer	Smad7	In a xenograft model study, stable overexpression of miR25 in colon cancer cells suppressed tumor growth.	[52]
miR-217	PDCA	KRAS	Xenograft tumors of PDAC cells were directly injected with miR217-expressing plasmids or a control vector using *in vivo*-jet PEI. The results from these assays indicated that miR-217 suppresses tumor cell growth *in vivo.*	[53]

Oncogenic miRNA: miR-221.

Tumor suppressive miRNA: miR-7, 520, 375, 25,217.

#### miR-221 as an oncogenic miRNA

miR-221 is one of the most frequently and consistently upregulated miRNAs in human cancer, including in HCC, pancreatic, colon, stomach, glioblastoma, kidney, bladder, prostate and thyroid cancer, which indicates its importance in tumorigenesis [48]. miR-221 has multiple gene targets, such as the cyclin-dependent kinase inhibitors p27Kip1 (CDKN1B/p27) [54, 55] and CDKN1C/p57 [55], the pro-apoptotic protein B-cell lymphoma 2-modifying factor (Bmf) [56], the inhibitor of the phosphoinositide 3-kinase pathway phosphatase and tensin homolog (PTEN) [57], the tissue inhibitor of metalloproteinase 3 (TIMP3) [57], the DNA damage- inducible transcript 4 (DDIT4), and a tumor suppressor that modulates the kinase activity of mammalian target of rapamycin (mTOR) [58].

Callegari *et al.* demonstrated that miR-221 could promote liver tumorigenicity in a transgenic mouse model of miR-221 overexpression in the liver. This model is characterized by the appearance of spontaneous liver tumors in a fraction of male mice and a strong acceleration of tumor development in 100% of mice treated with diethylnitrosamine (DEN). Ten-day old mice received one intraperitoneal injection of DEN, followed 2 months later by a single intravenous dose of anti-miR-221 oligonucleotide (AMO) diluted in saline solution every 15 days, for a total of three injections. Similar to human HCC, tumors in these mice were characterized by an increase in miR-221 expression and a concomitant inhibition of its target protein-coding genes (CDKN1B/p27 and Bmf, not CDKN1C/p57). As expected, mice that received an *in vivo* intravenous injection of anti-miR-221 oligonucleotides exhibited a significant reduction in the number and size of liver tumor nodules. This study not only shows that miR-221 can promote liver tumorigenicity, but it also establishes a valuable animal model for preclinical investigations of the use of anti-miRNA approaches to liver cancer therapy [48].

#### Tumor-suppressive miRNAs

Many studies have used the nude mouse xenograft model to evaluate the effects of a specific miRNA on tumorigenesis, tumor growth or metastasis *in vivo*. In particular, this model is commonly used to assess xenograft growth or progression after transplantation of cancer cells transfected with specific miRNA-expressing plasmids or empty vectors.

#### miR-7 in hepatocellular carcinoma (HCC) cells

miR-7 inhibits HCC cell growth and metastasis *in vitro* and *in vivo*. Phosphoinositide 3-kinase catalytic subunit delta (PIK3CD) was first identified as a miR-7 target, and further study suggested that miR-7 might be a key regulator of the PI3K/Akt/mTOR signaling pathway. In a xenograft model, overexpressed miR-7 effectively repressed tumor growth and decreased metastasis to the lung. These findings indicate that miR-7 functions as a tumor suppressor and plays a substantial role in inhibiting the tumorigenesis and reversing the metastasis of HCC through the PI3K/Akt/mTOR-signaling pathway. Given these results, miR-7 may be a potential therapeutic or diagnostic/prognostic target for treating HCC [49].

#### miR-520 in *hepatocellular carcinoma (HCC) cells*

The expression levels of miR-520e were decreased dramatically in HCC cells and clinical HCC tissues resulting from DNA hypermethylation in the upstream region of miR-520e locus, whereas silencing of the expression of miR-520e promoted cell proliferation [50]. Introduction of miR-520e suppresses the growth of HCC cells *in vitro* by targeting NF-κB-inducing kinase (NIK), which is involved in NIK/ERK/NF-κB signaling. To determine the effect of miR-520e on HCC cell growth *in vivo*, HepG2 cells transfected with miR-520e or a negative control were injected subcutaneously into nude mice. The introduction of miR-520e led to a significant reduction in both the size of tumor volume and the frequency of tumor formation. In addition, direct intratumoral injection with miR-520e oligonucleotides repressed the growth of HCC cells in an *in vivo* xenograft model. This finding provides new insight into the mechanism of hepatocarcinogenesis, indicating the therapeutic potential of miR-520e in the treatment of HCC [50].

#### miR-375 in hepatocellular carcinoma (HCC) cells

He *et al.* reported that miR-375 targets astrocyte elevated gene-1 (AEG-1) in HCC and suppresses liver cancer cell growth *in vitro* and *in vivo*. Overexpression of miR-375 in liver cancer cells decreased cell proliferation, clonogenicity, migration, and invasion, and induced G1 cell cycle arrest and apoptosis. Direct administration of cholesterol-conjugated 2’-O-methyl-modified miR375 mimics significantly affected the growth of HCC xenografts. These findings indicate that miR-375 targets AEG-1 in HCC and suppresses liver cancer cell growth [51].

#### miR-25 in colon cancer cells

Li *et al.* reported that miR-25 was significantly down-regulated in human colon cancer tissues, and identified Smad7 as its direct target. In a xenograft model study, stable overexpression of miR25 in colon cancer cells suppressed tumor growth [52]. These results suggest that miR-25 functions as a tumor suppressor by targeting Smad7 in colon cancer, suggesting that miR-25 may serve as a potential therapeutic target for colon cancer therapy.

#### miR-217 in pancreatic cancer cells

In most studies, cells overexpressing miRNAs or miRNA antagonists are used for xenografts and subsequent analyses. However, Zhao *et al.* reported another method of evaluating the function of miRNAs in vivo. They investigated the biological role of miR-217 in PDAC cells *in vitro* and *in vivo* since miR-217 is frequently down-regulated in pancreatic ductal adenocarcinoma (PDCA) [59, 60]. KRAS was identified as a direct target of miR-217 and, concordantly, up-regulation of miR-217 decreased KRAS protein expression and subsequently reduced the constitutive phosphorylation of downstream Akt. To confirm the function of miR-217 *in vivo*, xenograft tumors of PDAC cells were directly injected with miR217-expressing plasmids or a control vector using *in vivo*-jet PEI (Polyplus Transfection, Illkirch, France). The results from these assays indicated that miR-217 suppresses tumor cell growth *in vivo.* Therefore, miR-217 may serve as a therapeutic target for miRNA-based PDAC therapy [53].

### Development of effective delivery methods for miRNA-targeting oligonucleotides

The development of effective and safe delivery methods of miRNA-targeting molecules is critical to the success of miRNA-targeting drugs. Currently, modified oligonucleotides and various delivery particles are being used for these purposes (Table 3).

**Table 3:** **Comparison of systemic delivery methods**

Delivery method	Features	Advantage and disadvantage	Reference
AMOs	Complementary to mature miRNAs	AMOs are widely used to inhibit miRNAs *in vitro* and *in vivo*.	
Modified AMOs			
-OMe	2′-O-methyl modification	Modified AMOs have more stability and efficiency than AMOs.	[22],
			[61–63]
-MOE	2′-O-methoxyethyl modification	Especially LNA increases the stability, efficiency and specificity.	
-LNA	2′,4′-methylene modification		
Sponges	Competitive inhibitors which are transcripts expressed from plasmid with strong promoters, containing multiple, tandem binding sites to the miRNAs of interest.	Sponges can block a whole family of related miRNAs. Selectable marker or reporter gene in the vector allows to isolate a fraction of cells in which the family of miRNAs is strongly inhibited.	[64–67]
AAV	Adenovirus- associated vectors	AAV are also widely used for systemic delivery. While the toxicity of viral mediated delivery is rarely reported, it remains controversial.	[47], [62],
			[68]
(PEI/miR complex)	(Intratumoral injection)	PEI/miR complex, plasmid and CC9 are probably useful for delivery. However we cannot assure their utility because few experiments using them for delivery have been performed.	[69]
Plasmid	miRNA-expressing plasmids encapsulated in small multilamellar cationic liposome (DOTAP/cholesterol)		[70]
CC9	A specific tumor-homing and -penetrating bifunctional peptide conjugated with oligonucleotides.		[47]

AMOs: anti-miRNA oligonucleotides.

LNA: locked nucleic acid.

Chemically modified anti-miRNA oligonucleotides (AMOs) complementary to mature miRNAs are widely used to inhibit miRNAs *in vitro* and *in vivo*. Effective AMOs typically have proprietary modifications such as 2′-O-methyl, 2′-O-methoxyethyl or 2′,4′-methylene (LNA) [61], which increases their stability, efficacy [62, 63], and specificity [22].

In addition to LNA modification, other oligonucleotide modifications to increase effectiveness and tolerability are under development. These include cholesterol-conjugated modified “antagomirs” [71, 72] and “miRNA sponges”, which are competitive inhibitors of small RNAs [64–67].

“miRNA sponges” are transcripts that contain multiple, tandem binding sites to the miRNAs of interest. When vectors encoding these sponges are transfected into cells, multiple miRNA targets can be suppressed simultaneously [64]; however, further experiments are necessary to demonstrate the utility of the sponge method *in vivo*.

In terms of delivery methods, adenovirus-associated vectors are used widely for systemic delivery [47, 62]. However, while the toxicity of viral-mediated delivery is rarely reported in *in vivo* studies [62, 68], it remains controversial. Novel nanoparticle-based delivery systems, which may be safer and more amenable [47] than viruses, are being developed. As mentioned earlier, the systemic injection of low-molecular-weight PEI/miRNA mimic complexes has proved useful for intratumoral delivery in xenograft models [69]. Additionally, miRNA-expressing plasmids encapsulated in small multilamellar cationic liposomes (DOTAP/cholesterol), have been reported as a useful delivery method in the mouse xenograft model [70].

AMOs such as LNA are currently the most promising targeted therapy method in terms of safety, stability and prominence. Continuing studies *in vitro* and *in vivo* are undoubtedly critical to discovery of simple, efficient, and safe delivery methods; this will facilitate the development of miRNA-targeting therapies for human disease.

## Conclusions

Along with recent discoveries of the diverse effects of miRNAs in biological systems, miRNA-mediated intervention is a promising avenue for the development of novel therapeutics against human diseases. In addition to the current success of anti-miR122 therapy against chronic hepatitis C and the ongoing studies of miR-34 mimics against liver cancers in human clinical trials, the results of preclinical studies will likely lead to human clinical trials in the near future. However, several important issues must be addressed if this knowledge is to be used effectively in clinical trials. These include the delivery method, improved oligonucleotide modification for delivery, and safety. Because of its relative novelty, the safety of oligonucleotide therapy is an important consideration. Since miRNAs generally have diverse effects by targeting multiple mRNAs, undesired outcomes, so called “off-target effects”, may be encountered even when a specific miRNA is targeted. Effectiveness should also be considered. Although most current trials target individual miRNAs, targeting multiple miRNAs simultaneously may be necessary because most are considered to function cooperatively [73]. Therefore, although our understanding of miRNAs has increased, further research is needed to transform this knowledge into effective therapeutics against human diseases.
